# An Examination of the Practice of Bloodletting in Mental Disorders

**Published:** 1854-09

**Authors:** 


*An Examination of the Practice of Bloodletting in Mental Disorders* By PLINY EARLE, M. D. New York: Sam'l S. and W. Wood. 1854

In this essay the various authorities, on mental disorders, are *brought into focus* on the single point of the propriety of bloodletting. It is rather a book of quotations than of original matter, yet, as its value depends upon the weight of evidence adduced on either side, it furnishes, in a small compass, a most valuable index to the opinions of American and European writers on insanity. As might be anticipated, among the hundreds of opinions quoted, the large majority are against the practice of bloodletting in insanity.

England furnishes most of the lovers of the lancet, while the only advocate in America is Rush. It seems to be the settled opinion of the more reliable authorities, that but few cases of insanity can be benefitted by the lancet. While a wide difference of opinion still exists as to the essential nature of insanity, one class considering it as usually a purely mental or functional disturbance, while another refers all cases to actual organic disease, it is remarkable that there is so little variety in the treatment. This peculiar feature is explained when we recollect how prevalent the idea of *rest* has become as the great remedy in all disease. Thus the physician who recognizes in insanity an organic lesion, confines his treatment to the same calm, soothing routine, as does the one who considers it purely pschycical. Both look to rest and inactivity of function as the leading idea of cure, and expect that mental quiet and cheerfulness will produce their sanitary effect upon the diseased organ.

The final conclusion of Dr. Earle's book, is that the propriety of bloodletting rests upon very feeble analogies, derived from other and dissimilar diseases; and that it is generally unnecessary and injurious, and always of very limited application.

The industry and research displayed by the author in this little volume, is deserving of the highest praise, and we have every reason to believe, from a limited acquaintance with mental disorders, that its conclusions are sound and reliable. H.

